# Flow-Based Dynamic Approach to Assess Bioaccessible Zinc in Dry Dog Food Samples

**DOI:** 10.3390/molecules25061333

**Published:** 2020-03-15

**Authors:** Bruno J. R. Gregório, Ana Margarida Pereira, Sara R. Fernandes, Elisabete Matos, Francisco Castanheira, Agostinho A. Almeida, António J. M. Fonseca, Ana Rita J. Cabrita, Marcela A. Segundo

**Affiliations:** 1 LAQV, REQUIMTE, Departamento de Ciências Químicas, Faculdade de Farmácia, Universidade do Porto, Rua de Jorge Viterbo Ferreira n 228, 4050-313 Porto, Portugal; bruno.jr.gregorio@gmail.com (B.J.R.G.); saraferns@sapo.pt (S.R.F.); aalmeida@ff.up.pt (A.A.A.); 2 LAQV, REQUIMTE, Instituto de Ciências Biomédicas de Abel Salazar (ICBAS), Universidade do Porto, Rua de Jorge Viterbo Ferreira n 228, 4050-313 Porto, Portugal; amargaridabp@gmail.com (A.M.P.); ajfonseca@icbas.up.pt (A.J.M.F.); arcabrita@icbas.up.pt (A.R.J.C.); 3 SORGAL, Sociedade de Óleos e Rações S.A., Estrada Nacional 109, Lugar da Pardala, 3880-728 S. João Ovar, Portugal; Elisabete.matos@sojadeportugal.pt; 4 Alltechaditivos—Alimentação Animal Lda., Parque de Monserrate, Av. Dr. Luis Sá n 9 - Arm. A, 2710-089 Abrunheira, Portugal; fcastanheira@Alltech.com

**Keywords:** bioaccessibility, dog food, dog nutrition, dynamic extraction, flow analysis, kinetic profile, zinc

## Abstract

This work proposes a simple and easy-to-use flow-through system for the implementation of dynamic extractions, aiming at the evaluation of bioaccessible zinc and the characterization of leaching kinetics in dry dog food samples. The kinetic profile of Zn extraction was determined by flame atomic absorption spectroscopy and the results were fitted in an exponential function (R^2^ > 0.960) compatible with a two first-order reactions model. Values of fast leachable Zn ranged from 83 ± 1 to 313 ± 5 mg of Zn per kg of sample, with associated rate constants ranging from 0.162 ± 0.004 to 0.290 ± 0.014 min^−1^. Similar results were observed compared to the static batch extraction. The percentage of bioaccessible Zn ranged from 49.0 to 70.0%, with an average value of 58.2% in relation to total Zn content. Principal component analysis regarding the variables fast leachable Zn, associated rate constant, total Zn, and market segment, has shown that 84.6% of variance is explained by two components, where the second component (24.0%) presented loadings only for the fast leachable Zn and associated rate constant. The proposed method is suitable for the fast evaluation (<1 h) of leaching kinetics and bioaccessibility in dry dog food.

## 1. Introduction

Zinc, an essential trace element for dogs, is a component of several metalloenzymes that influence the metabolism of carbohydrates, lipids, proteins and nucleic acids [1]. It is also important for cellular immunity, reproductive and skin function, and wound healing [2,3]. Nowadays, companion animals’ tutors look for high quality pet food, that ensure the required energy and nutrients for the healthy growth and life of pets [4]. For zinc in particular, the minimum recommended level in complete dog food is 7.20 mg per 100 g of dry matter for dogs which are three to seven years of age and 8.34 mg per 100 g of dry matter for dogs over seven years of age, with a higher level for puppies (10.00 mg per 100 g of dry matter) [5]. There is also a maximum legal limit established in the EU, corresponding to 22.7 mg per 100 g of dry matter [6].

In this context, during the development of a new compound feed for animals, it is not only important to consider the nutritional guidelines and the legal limits, but also to assess the bioaccessibility and bioavailability of elements like Zn, because their source and the composition of the matrix influences both factors [7,8,9,10,11,12]. Zinc can be present in dog food as free or inorganic zinc, and also complexed with amino acids (e.g., histidine, methionine, glutamate, and glycine) or with other low-molecular-weight organic molecules (e.g., citrate, ascorbate, picolinate, and propionate) [2]. Bioaccessibility refers to the concentration of the nutrient that is released from the food matrix to the gastrointestinal (GI) tract and is available for absorption [13], being considered the first step towards bioavailability (the fraction that reaches systemic circulation from the GI tract), also representing the maximum value that can be achieved [9,14]. This parameter can be evaluated by using in vivo or in vitro methods [15]. However, in vitro studies present several benefits, such as being faster, less laborious and inexpensive [16,17], without concerns on animal welfare or ethical issues [18]. Consequently, static [15,19,20,21,22,23] and dynamic [9,24,25,26] in vitro models have been developed to evaluate the bioaccessibility of metals in different types of solid samples, such as food, soil and incineration ashes.

Dynamic methods, where online continuous leaching occurs, have numerous advantages like (i) a minimal sample manipulation, which reduces contamination, (ii) less time required for each extraction [27], (iii) possibility of automation of the extraction procedure [28], (iv) mimicking of naturally-occurring processes that are dynamic and not static [29], (v) reduction of the readsorption effects of the element to the surface of the matrix [30], and (vi) study of the leaching kinetics [24], allowing the discrimination of fast and slow leachable fractions [25,31,32]. Moreover, the constant pumping of extraction solution through the sample drives the dissolution equilibrium to the right, thereby providing information about the maximum amount of bioaccessible analyte [33]. The information obtained is important to help manufacturers choose the best ingredients for their products, taking into account the current legislation and aiming at complying with these limits without compromising the nutritional requirements. This means the formulation of dog food with a high amount of bioaccessible Zn within the legal limits established for total Zn.

The present work reports the development of a new, simple and easy-to-use flow-through system for on-line continuous leaching experiments and its application to the evaluation of bioaccessible Zn in complete dry dog food samples. The results obtained were compared with the static batch procedure. The characterization of the leaching kinetics of this metal was also targeted.

## 2. Results and Discussion

### 2.1. Extraction Chamber Configuration

The initial configuration of the extraction chamber (EC) was based on the scheme proposed by Maia et al. [25], using two equal filters to build it. The results obtained showed poor repeatability, with variable extraction profiles for the same sample and an RSD > 40% for the total amount of zinc extracted (*n* = 4). Compared to the previous work, a smaller amount of sample (ca. 35 mg) was used here and the size of the sample particles was larger (0.5 vs 0.25–0.35 mm). Moreover, complete dog foods are composed of several ingredients (cereals, meat, fish, etc.), creating a heterogeneous product (Supplementary Information, Table S1) [34]. The association of these factors may explain the low repeatability obtained.

**Figure 1:**
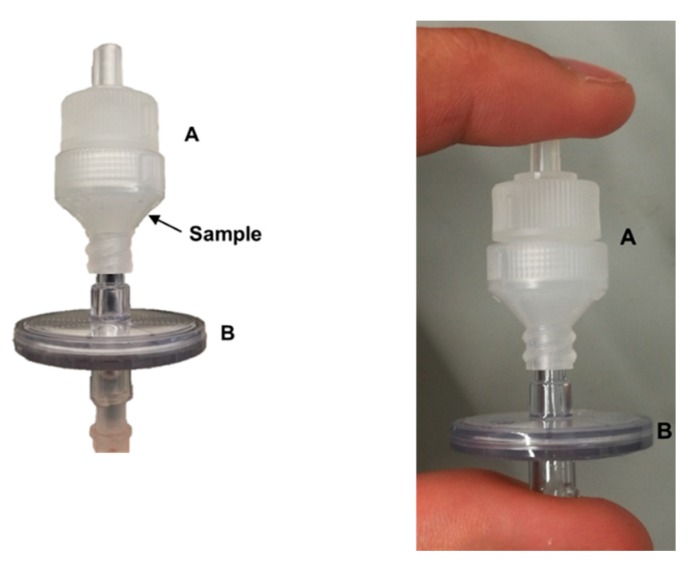
Extraction chamber (EC), composed by a polypropylene disk holder (**A**) with 25 mm of internal diameter containing a Fluoropore™ membrane filter (polytetrafluoroethylene), with a 1.0 µm pore, and by a Millex^®^ syringe filter (polyvinyl chloride housing and polyvinylidene fluoride membrane), with a 5.0 µm pore (**B**). Dry dog food sample is placed inside the EC, as indicated by the arrow, through the wide opening of the disk holder (Supplementary Information, Figure S1).

Since it was not possible to increase the mass of sample in the initial EC, the configuration was changed (Figure 1 and Supplementary Information, Figure S1a). In the new configuration, the sample was placed inside a larger polypropylene disk holder, through the wider opening (Supplementary Information, Figure S1b), and kept in place by the Millex^®^ syringe filter (Figure 1). Initially, 35 mg of sample were placed inside the holder, but non-reproducible results were still obtained, showing variable extraction profiles and RSD > 25% for the total amount of Zn extracted. Then, the amount of sample was increased to 70 mg, so that the lower part of the chamber was filled with it. The results obtained showed good repeatability regarding both the extraction profile and extracted zinc values (RSD < 2%, *n* = 2), due to the decrease of the void volume of the EC. This situation has been observed previously using other extraction chambers when the void volume was filled with a solid diluent (e.g., cellulose) [25]. This new configuration is simple, allowing the use of larger amounts of sample, and the disk holder can be reused in the following experiments, just having to be cleaned with the replacement of the filter membrane.

### 2.2. Study of the Extraction Procedure

The initial approach for the static batch protocol simulated the digestion in the stomach, followed by the digestion in the intestine. Using this approach, it was not possible to detect any amount of Zn after the gastric phase, since it precipitates in a pH > 6.0, as zinc oxide and/or zinc hydroxides, as reported elsewhere [35], and it was removed by the centrifugation and filtration steps before FAAS analysis. Hence, the following experiment mimicked only the gastric extraction phase, where it was possible to quantify Zn in the solution (Table 1). To demonstrate this rationale, one of the samples was subjected to both gastric and intestinal extractions and, before the centrifugation step, the extract was acidified to pH 2.0 with concentrated HCl. This way, the precipitated Zn was solubilized again, and its quantification was feasible, attaining similar results. Thus, in the following dynamic extraction experiments, only the gastric extraction procedure was performed.

In order to evaluate the bioaccessibility of Zn, the first step was to study the flow rate of the extraction solution. Flow rates of 0.5, 0.75 and 1.0 mL min^−1^ were tested. The experiment with 1.0 mL min^−1^ led to leakage of the extraction fluid, due to excessive backpressure in the system. The flow rates of 0.5 and 0.75 mL min^−1^ showed a similar extraction profile (Supplementary Information, Figure S2). Consequently, the flow rate of 0.5 mL min^−1^ was chosen to proceed with sample analysis.

The static batch protocol included the use of the gastric enzyme pepsin [36], so its influence in the dynamic extraction procedure was also assessed (Supplementary Information, Figure S3). The results obtained showed that the difference in the amount of Zn extracted in the presence or absence of pepsin is not statistically significant (paired t-test, *p* > 0.05, *n* = 4). The extraction protocol using pepsin was more complex, longer, and entailed the use of more reagents, leading to fluid leakages and increased system backpressure. So, as the results were similar, this enzyme was not included in the dynamic extraction protocol.

### 2.3. Zinc Bioaccessibility Assessment

With the purpose of evaluating the applicability of the proposed flow-based dynamic extraction scheme towards the assessment of bioaccessible Zn from dog food and the characterization of the extraction kinetics, three dry dog food samples of different market segments were analyzed via both dynamic and static methods. The results obtained are shown in Table 1. The relative difference values of Zn per kg found for the tested samples after 30 min of extraction ranged from 6% to 16% when compared to gastric batch extraction. Hence, the results have shown that the proposed procedure provided an alternative evaluation of the bioaccessible Zn when compared to the longer and more complex static batch extraction. The dynamic approach also brings the opportunity to perform the extraction in non-exhaustive conditions, as the extraction fluid is constantly renovated.

**Figure 2:**
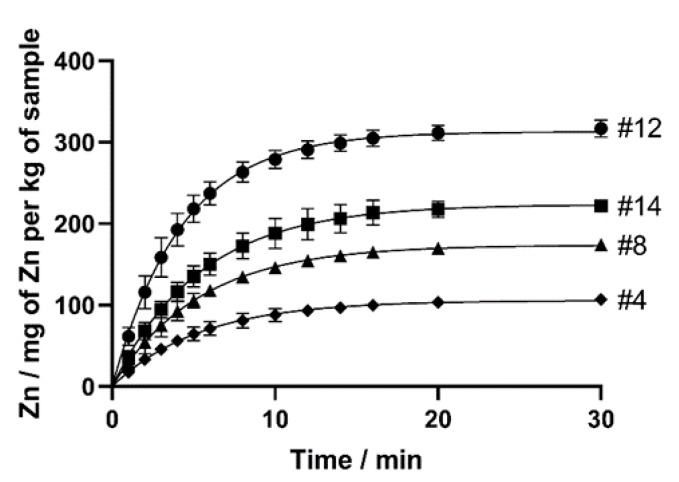
Kinetic profiles of bioaccessible Zn obtained for samples #4, #8, #12, and #14. Sample number is adjacent to the respective curve.

Then, a total of 14 dry dog food samples were tested by the dynamic extraction procedures, with the results obtained for 4 different samples shown in Figure 2. The extraction profiles of all samples, divided by market segment, are shown in Figure S4 of the Supplementary Information.

The kinetic extraction provides two types of information: (i) the bioaccessibility of the metal and (ii) the kinetics of the metal leaching [32]. The cumulative amount of bioaccessible Zn extracted at time t (C(t), mg of Zn per kg of sample) is showed to fit an exponential function C(t) = A × (1 − e^−Bt^) (Table 2, R^2^ > 0.960 for all experiments). This model is in good agreement with the two first-order reactions model reported by other authors [31,32], for readily extractable compounds. In this mathematical model, A represents the fast-leachable amount of Zn in the sample, and B is the associated rate constant. The values obtained for all tested samples are shown in Table 2.

Values of fast leachable Zn ranged from 83 ± 1 to 313 ± 5 mg per kg of sample, with associated rate constants ranging from 0.162 ± 0.004 to 0.290 ± 0.014 min^−1^. Globally, 77.1% to 91.5% of leachable Zn was released in the first 10 min of the experiment, showing a fast bioaccessibility in gastric acidic media. Good precision was attained, with RSD values ranging from 0.6% to 2.8% for the values of fast leachable Zn and from 1.7% to 5.9% for the associated rate constants.

**Figure 3:**
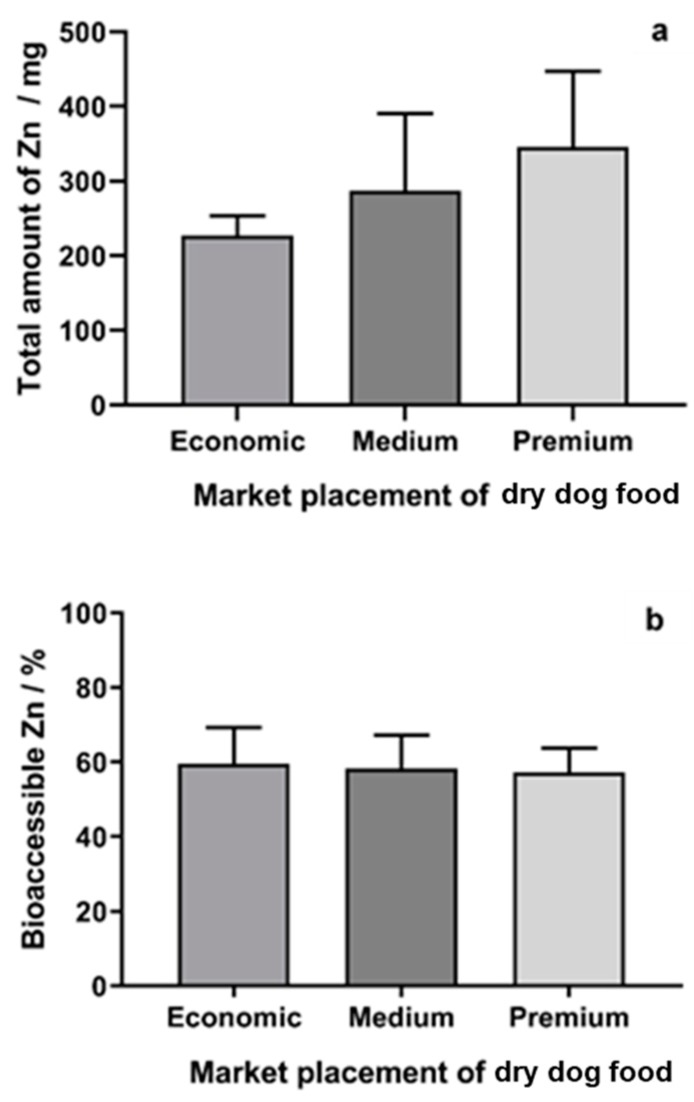
Total (**a**) and bioaccessible (**b**) Zn found in dry dog foods regarding their market segment. Economic dry dog food, *n* = 4; Medium type dry dog food, *n* = 4; Premium dry dog food, *n* = 6.

The total amount of Zn in samples ranged from 169 ± 9 to 526 ± 14 mg per kg, while the percentage of bioaccessible Zn ranged from 49.0% to 70.0%, with an average of 58.2%, providing values that show a good release of Zn from the solid matrix. When considering the results by market segment, the content of Zn in economic dry dog food varied from 204 ± 10 to 263 ± 36 mg per kg of sample, from 169 ± 9 to 421 ± 30 mg per kg of sample in medium type dry dog food and, in premium dry dog food, from 237 ± 10 to 526 ± 14 mg per kg of sample. The average bioaccessible Zn in economic dry dog food varied from 50.4% to 70.0% (average of 59.6%), from 49.0% to 69.4% (average of 58.2%) in medium type dry dog food, and from 50.3% to 68.1% (average of 57.3%) in premium dry dog food. For both evaluations (total Zn and bioaccessible Zn), the differences were not statistically significant between market segments (one-way ANOVA, *p* > 0.05) (Figure 3). However, the average results suggest a higher amount of total Zn in the premium segment, which will provide an absolute higher amount of bioaccessible Zn, because of the similar percentage of leachable Zn in the samples of the different market segments. Therefore, more samples must be analyzed to validate this rationale.

Moreover, considering the EU legal limit [6], most of the samples presented total Zn levels higher than the established limit (22.7 mg per 100 g of dry matter). This has also been reported for products present in the UK market, even considering the less strict previous legal limit (28.4 mg per 100 g of dry matter) [37]. Nevertheless, the EU legal limit is not surpassed if one considers the bioaccessible Zn content, for which only one sample (#7) was above it.

PCA was performed considering the variables A, B, total amount of Zn, and market segment. The Kaiser–Meyer–Olkin measure of sampling adequacy was 0.580, and *p* < 0.05 was obtained for the Bartlett’s test of sphericity. The total variance explained by the principal components, the component matrix, and the scree plot are given in the Supplementary Information Tables S2 and S3, and Figure S5, respectively. Two components were extracted (eigenvalue > 0.95) with loadings in all variables in the first component (explained variance of 60.6%), with higher values for the total and for the bioaccessible Zn. In the second component (explained variance of 24.0%), loadings were obtained only for variables A (bioaccessible zinc) and B, showing that this component depended only on the dynamic bioaccessibility features.

## 3. Materials and Methods

### 3.1. Chemicals and Solutions

All reagents used were of analytical reagent grade with no further purification. Ultrapure water (resistivity 18.2 MΩ cm) from arium^®^ water purification systems (Sartorius, Göttingen, Germany) was used for the preparation of all solutions. Sodium hydrogen phosphate dihydrate, sodium acetate, pepsin from porcine gastric mucosa (ref. P7012), pancreatin from porcine pancreas (ref. P1750), chloramphenicol and bovine bile (ref. B3883) were purchased from Sigma-Aldrich (St. Louis, MO, USA). Sodium dihydrogen phosphate monohydrate was acquired from Merck (Darmstadt, Germany). Sodium hydroxide and acetic acid were obtained from VWR (Fontenay-sous-Bois, France). Hydrochloric acid 37% (*w*/*w*) was acquired from Fisher Scientific (Leicestershire, UK). Nitric acid ≥ 69% (*w*/*w*) was acquired from Fluka (Seelze, Germany). Zinc standard solution (1000 mg L^−1^) was purchased from SCP Science (Courtaboeuf, France).

The extraction solution used in the dynamic procedure, mimicking only the gastric phase of digestion [36], was prepared by mixing of 25 mL of 0.1 M sodium phosphate buffer (pH 6.0) with 10 mL of 0.2 M HCl and adjusting the pH to 2.0 with 1 M HCl or 1 M NaOH.

For the static batch extraction protocol, a gastric phase solution was prepared by mixing 25 mL of 0.1 M sodium phosphate buffer (pH 6.0) with 10 mL of 0.2 M HCl and by adjusting the pH to 2.0 with 1 M HCl or 1 M NaOH. Then, 1 mL of 0.1 mg mL^−1^ chloramphenicol solution (10 mg in 100 mL of 50 mM sodium acetate buffer) and 1 mL of freshly prepared 14 mg mL^−1^ pepsin solution (14 mg in 1 mL of 0.1 M HCl) were added to the previous solution. For the surrogate intestinal juice, added after the gastric digestion, 10 mL of 0.2 M phosphate buffer (pH 6.8) and 5 mL of 0.6 M NaOH were added to the flask. After adjusting the pH to 6.8, 1 mL of freshly prepared 100 mg mL^−1^ pancreatin solution (100 mg in 1 mL of 0.2 M sodium phosphate buffer) and 1 mL of freshly prepared 25 mg mL^−1^ bile solution (25 mg in 1 mL of 0.2 M sodium phosphate buffer) were added to the previous solution.

### 3.2. Samples

Fourteen complete dry dog food samples from several market segments (economic, medium and premium) were acquired from local supermarkets, specialized stores and veterinary clinics. Market segment was attributed regarding their price per kg: ≤1.20 €, ≤5.30 €, and >5.30 €, for economic, medium and premium dry dog food, respectively. They were ground and passed through a 0.5 mm pore sieve to standardize the size of solid food particles [15] and stored in plastic containers. Prior to the analysis, they were dried for a period of 24–72 h in a laboratory oven at 65 °C.

### 3.3. Flow-Based Dynamic Extraction Apparatus

**Figure 4:**
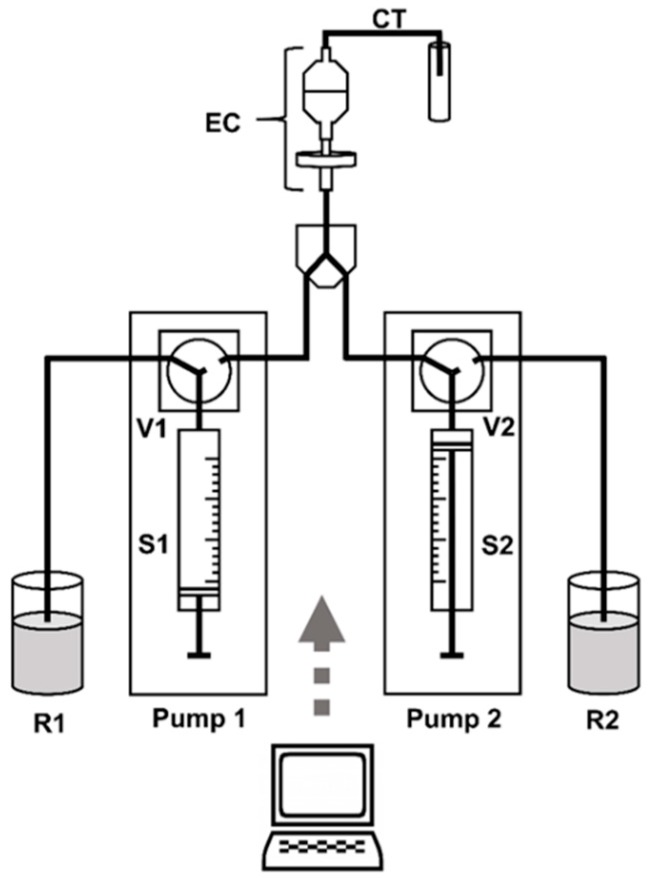
Schematic representation of the flow-based dynamic extraction apparatus. S1 and S2, syringes; V1 and V2, solenoid valves; EC, extraction chamber; CT, collecting tube; R1 and R2, extraction fluid reservoirs.

Two syringe pumps (Multi-Burette 4S, Crison Instruments, Allela, Spain) working in tandem were used to propel the extraction solution through the system. The tandem configuration permitted for one syringe to fill while the other dispensed the solution, therefore creating a continuous flow, achieving a dynamic extraction. Pump 1 was equipped with a 5 mL syringe and pump 2 with a 2.5 mL syringe, being supplied by reservoirs 1 and 2, respectively, with extraction solution. Solenoid commutation valves, V1 and V2, allowed the connection of the syringes to the extraction chamber (EC) (position on) or to the reservoir (position off). A Y-shaped confluence was used to connect the valves to the EC. Polytetrafluorethylene tubing with 0.8 mm of internal diameter (Omnifit, Cambridge, UK) was used for all connections (Figure 4). The number of steps, the velocity and the direction of movement of the syringe pumps, and the position of the solenoid valves were controlled by a personal computer running a lab-made software written in QuickBasic 4.5 (Microsoft, Redmond, WA, USA).

### 3.4. Extraction Chamber

The extraction chamber proposed is shown in Figure 1. It is a combination of a polypropylene disk holder with 25 mm of internal diameter, containing a Fluoropore™ membrane filter (made from polytetrafluoroethylene, pore size of 1.0 µm), with a Millex^®^ syringe filter (constituted by a polyvinyl chloride housing and a polyvinylidene fluoride membrane, pore size of 5.0 µm), both acquired from Merck. The sample was placed inside the polypropylene disk holder through the wide opening (Supplementary Information, Figure S1) and kept in place with the second filter. The extraction solution flowed from the Millex^®^ syringe filter to the disk holder, with an upwards movement, ensuring that all sample particles are in constant contact with the extractant. An adaptation of the system proposed by Maia et al. [25], using two Millex^®^ syringe filters, was also tested.

### 3.5. Flow-Based Dynamic Extraction Procedure

Before the leaching procedure started, the syringes and all tubing upstream the EC had to be washed and filled with extraction solution. After this, the EC containing the previously weighed sample was connected to the system and to the collecting tube (Figure 4). When the system was ready, an instruction was given by computer control for the experiment to begin and S1 started to dispense its content. When 90% of S1 was dispensed, S2 was activated and dispensed the solution to the reservoir (off position). As soon as S1 was emptied, V2 was commuted to the position on, and the extraction solution went into the system and through the EC. When one of the syringes was dispensing its content, the other was refilling, working in tandem, thus maintaining a continuous flow that was crucial for the kinetic study of the leaching procedure.

The sampling scheme was chosen in order to provide the most detailed information possible about the extraction kinetic profile, without compromising the total time of the assay and reagent consumption. Hence, 13 fractions were collected during the procedure, employing a 0.5 mL min^−1^ flow rate. The first 6 fractions were collected every minute (0.5 mL each), so more information about the extraction process was acquired in the first minutes of leaching. The following 5 every 2 min (1.0 mL each), the 12th after 4 min (2.0 mL) and the last (5.0 mL) after 10 min (Supplementary Information, Table S4). Other variables were studied in a univariate fashion, considering the detection limit of the Zn quantification method and the maximum pressure sustained by the system before leakage occurs.

### 3.6. Static Batch Extraction Procedure

The static extraction method was based on the method described by Hervera et al. [36]. One gram of sample was weighed into a 50 mL Erlenmeyer flask, and the gastric extraction solution was added to the flask. The flask was then closed and, by using a thermostatically controlled water bath at 39 °C, its content was incubated for 2 h with constant magnetic stirring. After incubation, the flasks were cooled, following the addition of surrogate intestinal juice (please see Chemicals and Solutions). Then, the flask was incubated again for 4 h at 39 °C, with constant stirring. The final step was centrifugation (2 × 30 min at 4713 g, Allegra X-15R, Beckman Coulter, Indianapolis, IN, USA), followed by filtration of the supernatant using a FilterBio^®^ polytetrafluorethylene syringe filter, with a pore size of 0.45 µm (Filter-Bio, Jiangsu, People’s Republic of China).

### 3.7. Determination of Zinc and Leaching Profile

The total amount of Zn in the samples was determined by inductively coupled plasma mass spectrometry as described by Pereira et al. [3], using an iCAP Q™ (Thermo Fisher Scientific, Schwerte, Germany) instrument, equipped with a MicroMist™ nebulizer, a Peltier cooled cyclonic spray chamber, a standard quartz torch and nickel skimmer and sampling cones. Operation conditions were: RF power 1550 W; auxiliary Ar flow rate 0.80 L min^−1^; nebulizer flow rate 1.08 L min^−1^ and plasma flow rate 14 L min^−1^. High purity (99.9997%) Ar (Gasin II, Leça da Palmeira, Portugal) was used as the nebulizer and plasma gas. Zn was determined as ^66^Zn isotope.

Zinc present in the fractions collected during the flow-based dynamic extraction procedure was measured through flame atomic absorption spectroscopy (FAAS), using an AAnalyst 200 instrument (PerkinElmer, Überlingen, Germany). A hollow cathode lamp (PerkinElmer) was used as the radiation source. The flame was an air-acetylene mixture, using 10.0 L min^−1^ oxidant flow and 2.5 L min^−1^ acetylene flow.

For calibration, a matrix-matching approach was implemented. First, several standard stock solutions with concentrations ranging from 0.25 to 7.0 mg L^−1^ of Zn, containing 2.0% (*v*/*v*) of nitric acid, were prepared. Then, calibration standard solutions were prepared by mixing 300 µL of the previous solutions with 400 µL of the gastric extraction juice, and the volume was completed to 3.0 mL with water (1:10 dilution). For the sample analysis, 400 µL of each fraction was mixed with 6 µL of nitric acid and diluted in water to a final volume of 3.0 mL (final concentration of nitric acid of 0.2% (*v*/*v*)). Blank samples were prepared alongside the test samples by replacing the extracted fraction by gastric extraction juice, and they were used for the correction of the analytical signals.

Absorbance values were measured at a wavelength of 213.86 nm. The values obtained for samples were directly interpolated in the calibration curve of matrix matched Zn standards (mg L^−1^). The content of Zn in mg per kg was calculated considering the volume of the fraction collected, V (µL) (Supplementary Information, Table S4), and the mass of sample weighed into the disk holder, m(mg), as follows: [Zn] = [(Abs − Intercept) × Dilution factor × V (µL)] / [Slope × m (mg)]. To define the kinetic profile of the extraction process, Zn content was plotted as the cumulative leached value (mg of Zn per kg of sample) as a function of time. Each dry dog food sample was analyzed in two independent experiments, comprising the collection of 13 fractions in each experiment (total number of sampling points was 26). For each sampling point, FAAS analysis was performed in triplicate, totalizing a number of 78 readings for each curve fitting. Precision was estimated as the relative standard deviation, calculated from the standard error and the mean value obtained for parameters A and B after curve fitting to the exponential function C(t) = A × (1 − e^−Bt^).

### 3.8. Statistical Analysis

The comparison of the mean value of Zn extracted in the flow-based dynamic extraction using (or not) pepsin was performed by applying a paired t-test. The comparison of the mean value of bioaccessible and total amount of Zn between samples of different market segments was done by applying a one-way ANOVA. The characterization of the leaching kinetics was done by fitting the data via nonlinear regression using a first order mathematical model. These operations were completed using GraphPad Prism 7 software (GraphPad Software, San Diego, CA, USA). Principal component analysis (PCA) was performed using IBM SPSS Statistics 26 software (IBM, Armonk, NY, USA), with a maximum of 25 iterations for convergence for matrix extraction and no rotation.

## 4. Conclusions

The dynamic extraction protocol proposed here provides a simple, fast and accurate assessment of bioaccessible Zn from dry dog food samples and their leaching kinetics, including both free and molecule-associated forms. This procedure has several advantages when comparing to traditional static batch methods, with fewer steps and more flexibility, as the combination of number of fractions and collection time can be changed, in order to perform the intended characterization of the extraction profile. Additionally, dynamic methods work on non-exhaustive conditions, since the extraction solutions are continuously propelled through the sample, driving the dissolution equilibrium to the right, providing information about the worst-case scenario, and not under equilibrium conditions that do not mimic naturally occurring processes.

The total amount of Zn in samples ranged from 169 ± 9 to 526 ± 14 mg per kg, while the percentage of bioaccessible Zn ranged from 49.0 to 70.0%, with an average of 58.2%. Similar results for bioaccessible Zn were obtained for samples tested using the proposed method and the time-consuming batch method. Moreover, most of the samples presented total Zn levels higher than the EU legal limit, but this value was not surpassed if the bioaccessible Zn content is considered (except for one sample). Despite the limited number of analyzed samples, the average results suggest a higher amount of total Zn in the premium segment, which will provide an absolute higher amount of bioaccessible Zn, because of the similar percentage of leachable Zn in the samples of the different market segments.

Finally, the reusable disk holder is an important improvement compared to previous flow systems proposed for bioaccessibility assessment, since it contributes to a more environmentally sustainable and cost-effective analysis. The extraction chamber proposed could also be utilized to perform extractions of other components in different products, such as other solid food materials, and environmental and pharmaceutical solid samples.

## Figures and Tables

**Table 1:** Values of bioaccessible Zn ^1^ using the flow-based dynamic extraction and the static batch extraction.

Sample *^2^*	Dynamic Extraction	Batch Extraction	
		Gastric	Gastric + Intestinal
Sample #A	107 ± 5	102 ± 7	ND
Sample #B	224 ± 4	194 ± 3	ND
Sample #C	222 ± 4	210 ± 9	ND

^1^ Values expressed as mg of Zn per kg of sample, *n* = 2; ^2^ Economic dry dog food (Sample #A), medium type dry dog food (Sample #B), and premium dry dog food (Sample #C); ND, Not detected.

**Table 2:** Cumulative Zn obtained experimentally, parameters A (fast leachable amount) and B (associated rate constant) ^1^, obtained after fitting of experimental data to the kinetic equation C(t) = A × (1 − e^−Bt^), total amount of Zn obtained by inductively coupled plasma mass spectrometry, % of bioaccessible Zn, and market segment of the tested samples.

Sample	Cumulative Bioaccessible Zn/mg kg^−1^	A/mg kg^−1^	B/min^−1^	Total Amount of Zn/mg kg^−1^	Bioaccessible Zn/%	Market Segment
Sample #1	186 ± 4	176 ± 3	0.290 ± 0.014	352 ± 40	52.8	Premium
Sample #2	158 ± 1	160 ± 4	0.178 ± 0.010	295 ± 8	53.5	Premium
Sample #3	120 ± 2	118 ± 2	0.163 ± 0.004	230 ± 57	52.3	Economic
Sample #4	83 ± 1	83 ± 1	0.255 ± 0.008	169 ± 9	49.0	Medium
Sample #5	161 ± 2	161 ± 1	0.236 ± 0.004	237 ± 10	68.1	Premium
Sample #6	143 ± 0	149 ± 2	0.162 ± 0.004	204 ± 10	70.0	Economic
Sample #7	317 ± 10	313 ± 5	0.233 ± 0.010	526 ± 14	60.2	Premium
Sample #8	144 ± 1	143 ± 2	0.211 ± 0.007	286 ± 10	50.3	Premium
Sample #9	191 ± 3	189 ± 3	0.182 ± 0.008	275 ± 5	69.4	Medium
Sample #10	174 ± 1	174 ± 3	0.187 ± 0.008	285 ± 15	61.2	Medium
Sample #11	222 ± 4	224 ± 5	0.184 ± 0.010	378 ± 23	58.7	Premium
Sample#12	173 ± 3	160 ± 3	0.262 ± 0.013	263 ± 36	65.7	Economic
Sample #13	224 ± 4	215 ± 3	0.239 ± 0.009	421 ± 30	53.3	Medium
Sample #14	107 ± 5	106 ± 3	0.185 ± 0.011	212 ± 18	50.4	Economic

^1^ R^2^ > 0.960, *n* = 26 from two independent experiments.

## Supplementary Materials

The following are available online. Table S1. Main ingredients present on the tested dry dog food samples according to label information, Table S2. Total variance explained obtained by principal component analysis (PCA), Table S3. Component matrix obtained after the extraction method of the PCA, Table S4. Fraction collection time, fraction volume and total extraction volume for the extraction procedure, Figure S1. (a) Extraction chamber. A and D, polypropylene disk holder; B, O-ring; and C, Fluoropore™ membrane filter (polytetrafluoroethylene) with a 1.0 µm pore. (b) After the assembly of all parts, sample is placed inside the A moiety, through its wider opening, Figure S2. Kinetic profiles of bioaccessible Zn obtained for the dynamic extraction using flow rates of 0.5 mL min^−1^ and 0.75 mL min^−1^, Figure S3. Comparison of the extraction profiles with and without pepsin, *n* = 2, Figure S4. Kinetic profiles of bioaccessible Zn obtained for all 14 samples, representing different types of market segment: (a) economic dry dog food, (b) medium type dry dog food, and (c) premium dry dog food, *n* = 2, Figure S5. Scree plot obtained by PCA.
