# Supplementary material for: Flow-Based Dynamic Approach to Assess Bioaccessible Zinc in Dry Dog Food Samples
Source: Molecules. 2020 Mar 15;25(6):1333. doi: 10.3390/molecules25061333 (PMC7144096; doi:10.3390/molecules25061333)
Supplement: Supplementary file 1 [file molecules-25-01333-s001.pdf]

Electronic Supplementary Information

Flow-based dynamic approach to assess bioaccessible zinc in dry dog food samples

Bruno J. R. Gregório,[1] Ana Margarida Pereira,[2] Sara R. Fernandes,[1] Elisabete Matos,[3] Francisco Castanheira,[4] Agostinho A. Almeida,[1] António J. M. Fonseca,[2] Ana Rita J. Cabrita,[2] Marcela A. Segundo,[1*]

[1] LAQV, REQUIMTE, Departamento de Ciências Químicas, Faculdade de Farmácia, Universidade do Porto, Rua de Jorge Viterbo Ferreira nº 228, 4050-313 Porto, Portugal

[2] LAQV, REQUIMTE, Instituto de Ciências Biomédicas de Abel Salazar (ICBAS), Universidade do Porto, Rua de Jorge Viterbo Ferreira nº 228, 4050-313 Porto, Portugal

[3] SORGAL, Sociedade de Óleos e Rações S.A., Estrada Nacional 109, Lugar da Pardala, 3880-728 S. João Ovar, Portugal

[4] Alltechaditivos – Alimentação Animal Lda., Parque de Monserrate, Av. Dr. Luis Sá nº 9 - Arm. A, 2710-089 Abrunheira, Portugal

*Corresponding author.

E-mail: msegundo@ff.up.pt

Tel: +351 220428676

Table S1. Main ingredients present on the tested dry dog food samples according to label information.

Sample #	Main ingredients
1	Cereals (maize starch and rapeseed flour) and derivatives of vegetable origin (pea protein).
2	Meat and animal derivatives (dried poultry protein) and cereals (maize and rice).
3	Cereals (rice > 15%), meat and animal derivatives, and derivatives of vegetable origin (vegetable protein extracts).
4	Cereals (ground maize), meat and animal derivatives (poultry > 4%), and oils and fats.
5	Cereals (> rice), cereals (maize protein), and fish and fish derivatives (fish meal).
6	Cereals, meat and animal derivatives (meat flour), and vegetables (tubers and roots).
7	Fish and fish derivatives (salmon), vegetables (sweet potato), and oils and fats (chicken fat).
8	Cereals (maize), derivatives of vegetable origin (cellulose), and meat and animal derivatives (dried chicken and turkey protein).
9	Cereals (53% whole grain cereals), meat and animal derivatives (chicken > 4%), and vegetables.
10	Cereals (64% whole grain cereals), meat and animal derivatives (beef > 4%), and vegetables.
11	Cereals (wheat and oats), fish and fish derivatives (fish meal), and derivatives of vegetable origin (cellulose).
12	Cereals (15% wheat and 15% maize), meat and animal derivatives (10% chicken), and derivatives of vegetable origin (4% peas).
13	Cereals (4%rice), meat and animal derivatives (4% chicken), and oils and fats.
14	Cereals, meat and animal derivatives, and derivatives of vegetable origin.

Table S2. Total variance explained obtained by principal component analysis (PCA).

	Initial Eigenvalues		
**Component**	**Total**	**% of Variance**	**Cumulative %**
1	2.423	60.572	60.572
2	0.958	23.961	84.533
3	0.560	14.007	98.540
4	0.058	1.460	100.000

Table S3. Component matrix obtained after the extraction method of the PCA.

	Component	
	**1**	**2**
Total amount of Zn	0.943	n/a
A	0.904	-0.339
Market Segment	0.743	n/a
B	0.404	0.875

Table S4. Fraction collection time, fraction volume and total extraction volume for the extraction procedure.

Fraction #	Collection time[a] / min	Fraction volume[b] / mL	Total extraction volume / mL
1	1	0.5	0.5
2	2	0.5	1.0
3	3	0.5	1.5
4	4	0.5	2.0
5	5	0.5	2.5
6	6	0.5	3.0
7	8	1.0	4.0
8	10	1.0	5.0
9	12	1.0	6.0
10	14	1.0	7.0
11	16	1.0	8.0
12	20	2.0	10.0
13	30	5.0	15.0

[a] after complete filling of the collecting tube (CT) (Figure 4)

[b] flow rate of 0.5 mL min^{-1}

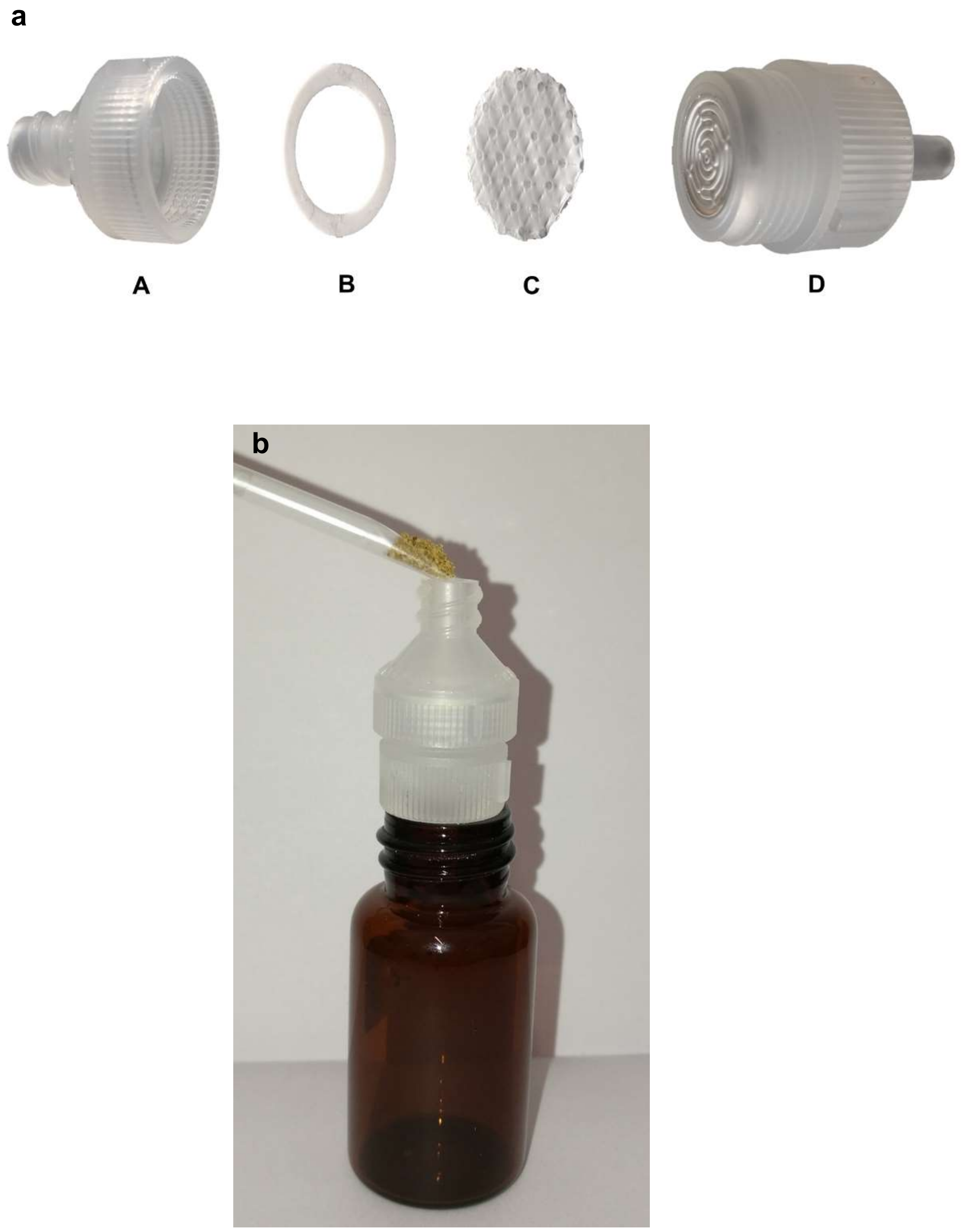

Figure S1. (a) Extraction chamber. A and D, polypropylene disk holder; B, O-ring; and C, Fluoropore™ membrane filter (polytetrafluoroethylene) with a 1.0 µm pore. **(b)** After the assembly of all parts, sample is placed inside the A moiety, through its wider opening.

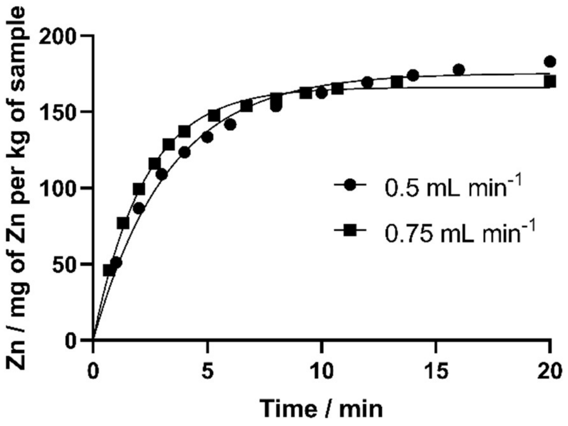

Figure S2. Kinetic profiles of bioaccessible Zn obtained for the dynamic extraction using flow rates of 0.5 mL min^{-1} and 0.75 mL min^{-1}.

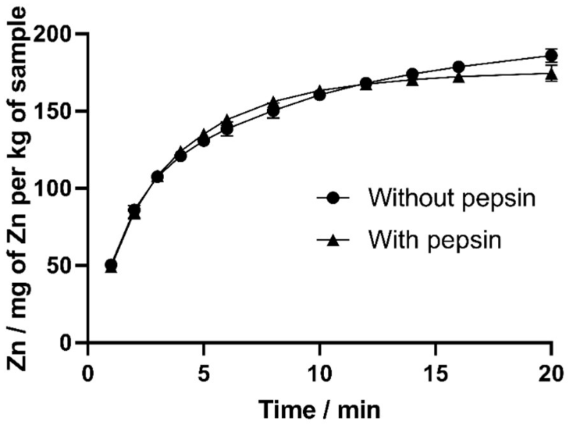

Figure S3. Comparison of the extraction profiles with and without pepsin, $n = 2$.

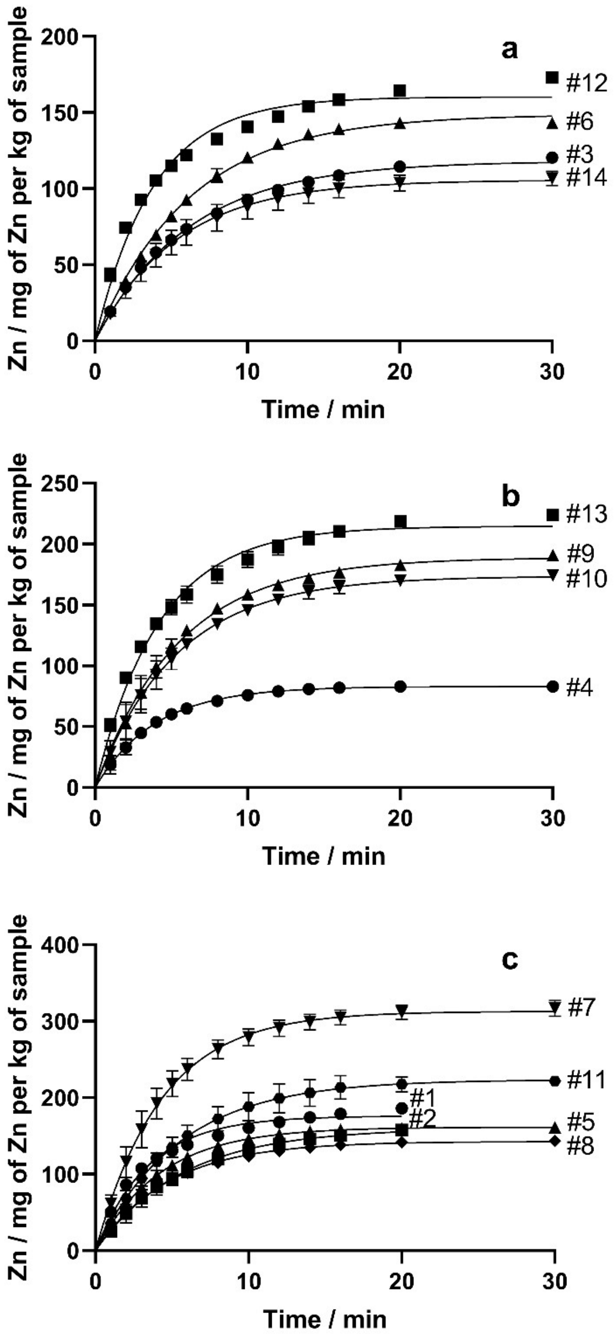

Figure S4. Kinetic profiles of bioaccessible Zn obtained for all 14 samples, representing different types of market segment: **(a)** economic dry dog food, **(b)** medium type dry dog food, and **(c)** premium dry dog food, *n* = 2. Samples #1 and #2 were subject to the extraction for 20 minutes.

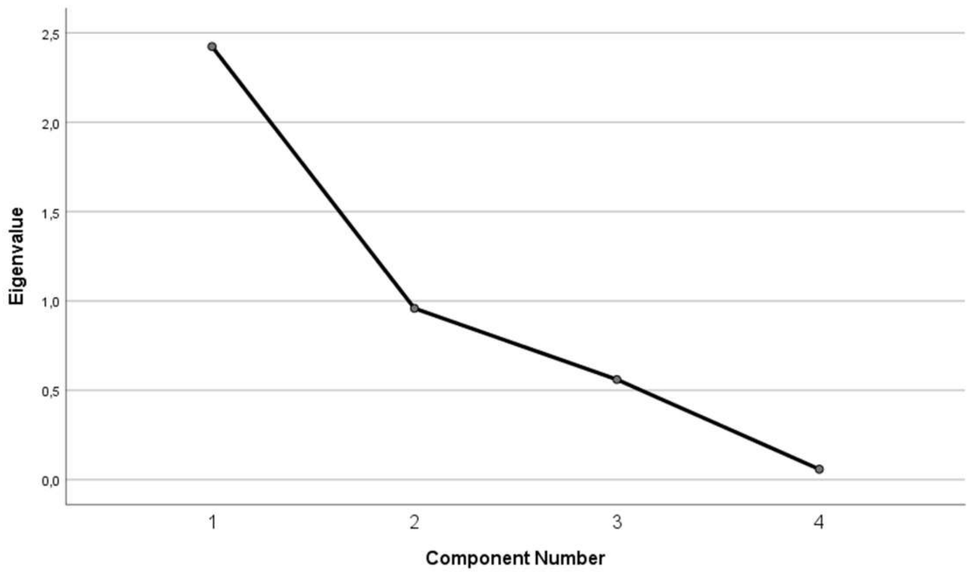

Figure S5. Scree plot obtained by PCA.
